# Implementation and Evaluation of a Risk-Stratified Nurse-Led Thyroid Cancer Follow-Up Clinic: A Single-Centre Retrospective Service Evaluation

**DOI:** 10.7759/cureus.105098

**Published:** 2026-03-12

**Authors:** Krishna Mohan Kaimal

**Affiliations:** 1 Otolaryngology - Head and Neck Surgery, Royal Blackburn Hospital, Blackburn, GBR

**Keywords:** head and neck oncology, nurse-led follow-up, outpatient service redesign, quality improvement, risk stratification, thyroid cancer

## Abstract

Background: Post-treatment surveillance for differentiated thyroid cancer (DTC) can extend for many years, contributing to increasing outpatient follow-up workload. Risk-stratified models that delegate selected low-risk follow-up to nurse-led clinics may improve sustainability if delivered within a robust governance framework.

Methods: We undertook a single-centre retrospective service evaluation in a UK district general hospital to assess the implementation and safety of a nurse-led, risk-stratified thyroid cancer follow-up pathway. The evaluation period was July 14, 2020, to February 13, 2026. Sixty eligible low-risk DTC patients were analysed as a random sample: 30 follow-up encounters managed in a consultant-led clinic and 30 managed in a nurse-led clinic. A structured follow-up proforma standardised documentation of red flag symptom screening, wound assessment, voice assessment, and biochemical review (including calcium/parathyroid hormone where clinically indicated), and defined escalation criteria to consultant review.

Results: In the nurse-led cohort, one patient required escalation for assessment of a submandibular swelling, with subsequent review confirming reactive lymphadenitis and return to the nurse-led pathway. The escalation proportion was 3.3% with a 95% confidence interval (CI) of 0.6%-16.7%. No missed malignancies, unplanned readmissions, or safety incidents were identified during the evaluation. Protocol compliance across safety domains in the nurse-led cohort was 100%.

Conclusion: A governance-supported nurse-led thyroid cancer follow-up clinic for appropriately selected low-risk DTC patients can be delivered safely, with high protocol compliance and low escalation rates, supporting sustainable outpatient service redesign.

## Introduction

Differentiated thyroid cancer (DTC) frequently has excellent long-term outcomes, but routine surveillance often continues for years, creating a cumulative outpatient follow-up burden as service caseloads rise. Contemporary management emphasises risk stratification and dynamic risk assessment to individualise surveillance intensity, enabling lower-intensity follow-up for patients with an excellent response to therapy and stable clinical and biochemical findings [1-3].

Guidance from the National Institute for Health and Care Excellence supports structured ongoing monitoring and follow-up for DTC [4], and broader cancer follow-up policy in NHS England promotes personalised stratified follow-up approaches to improve patient experience and use outpatient capacity efficiently [5].

Alternative follow-up models, including nurse-led clinics, are increasingly used across oncology, with qualitative evidence highlighting the importance of clear communication, patient reassurance, and accessible escalation routes [6]. In thyroid cancer specifically, published experience describes feasibility and patient acceptability when nurse-led follow-up is supported by appropriate specialist competencies and governance [7].

This manuscript reports a retrospective service evaluation of a locally implemented risk-stratified nurse-led thyroid cancer follow-up pathway, focusing on safety, escalation, and protocol compliance outcomes.

## Materials and methods

This was a single-centre retrospective service evaluation of a nurse-led thyroid cancer follow-up pathway implemented within head and neck oncology outpatient services at Royal Blackburn University Teaching Hospital, Blackburn. The evaluation was conducted to assess the standard achieved by this service in routine practice and was managed within local governance as a service evaluation rather than research. In line with UK governance distinctions defined by the Health Research Authority [8], service evaluation typically does not require NHS Research Ethics Committee review but remains subject to local clinical governance and information governance requirements.

The evaluation period was from July 14, 2020, to February 13, 2026 (study end date corresponding to the dataset closure used for analysis in this evaluation). The nurse-led clinic pathway was introduced during the evaluation period, enabling assessment of both consultant-led and nurse-led follow-up encounters within the same institution.

Eligible patients were adults (≥18 years) with histologically confirmed differentiated thyroid carcinoma who had undergone total thyroidectomy or completion thyroidectomy (with or without radioactive iodine, where applicable) and were categorised as low risk at follow-up using dynamic risk stratification principles. Dynamic risk stratification refers to the ongoing reassessment of recurrence risk based on clinical, biochemical, and radiological response to treatment, as described in established thyroid cancer follow-up frameworks. Patients typically demonstrated an excellent or indeterminate response to therapy according to dynamic risk stratification criteria used in differentiated thyroid cancer follow-up. Patients were excluded if they had intermediate- or high-risk disease, rising thyroglobulin, suspicious cervical lymphadenopathy, or structural recurrence, or required escalation for multidisciplinary discussion at the index follow-up.

A structured nurse-led follow-up proforma was used to guide and document consultations and to support standardisation of surveillance, safety screening, and escalation decisions. The data fields captured in the proforma are summarised in the Appendices, including biochemical markers (thyroglobulin, anti-thyroglobulin antibodies, and thyroid-stimulating hormone), ultrasound findings, dynamic risk category, escalation status, and 30-day re-attendance status. The operational data definitions used in the evaluation are clarified in the Appendices.

The primary outcome measures for this service evaluation were (i) protocol compliance with key safety domains in the nurse-led pathway, (ii) escalation rate from nurse-led follow-up to consultant assessment, and (iii) short-term safety outcomes, including unplanned readmission or re-attendance within 30 days. The secondary outcomes included descriptive service capacity indicators (e.g., appointment slots generated) and patient experience as recorded in routine service feedback/complaint outcomes. Patient experience feedback referenced in this evaluation reflects routine service feedback mechanisms rather than data derived from a formally validated questionnaire.

A total sample of 60 patients was analysed as a pragmatic random sample of eligible low-risk DTC follow-up encounters during the evaluation period, selected to provide balanced representation across clinic models (30 consultant-led and 30 nurse-led) for rapid safety and governance appraisal. No formal power calculation was undertaken because the evaluation aim was descriptive estimation of safety/process outcomes rather than hypothesis testing.

Statistical analysis used a descriptive approach. Categorical outcomes were summarised as counts and percentages. The nurse-led escalation proportion was reported with a two-sided 95% confidence interval (CI) calculated using the Wilson score method for a single proportion, selected because score-based intervals have improved coverage properties compared with simple Wald intervals, particularly with small numerators [9]. Analyses were performed using R statistical software version 4.3.3 (R Foundation for Statistical Computing, Vienna, Austria).

## Results

**Figure 1:**
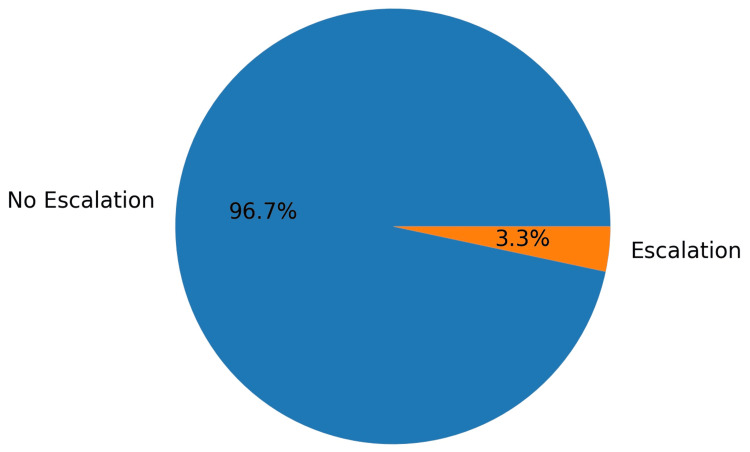
Escalation rate in nurse-led cohort (3.3%; 95% CI: 0.6%-16.7%) CI: confidence interval

Sixty patients were analysed (30 consultant-led and 30 nurse-led). In the nurse-led cohort, one patient required escalation for assessment of a submandibular swelling. Subsequent evaluation confirmed reactive lymphadenitis, and the patient returned to the nurse-led pathway. The escalation rate was 3.3% (95% CI: 0.6%-16.7%). A graphical representation of escalation versus non-escalation in the nurse-led cohort is shown in Figure 1.

No missed malignancies, unplanned readmissions, or safety incidents were recorded.

**Figure 2:**
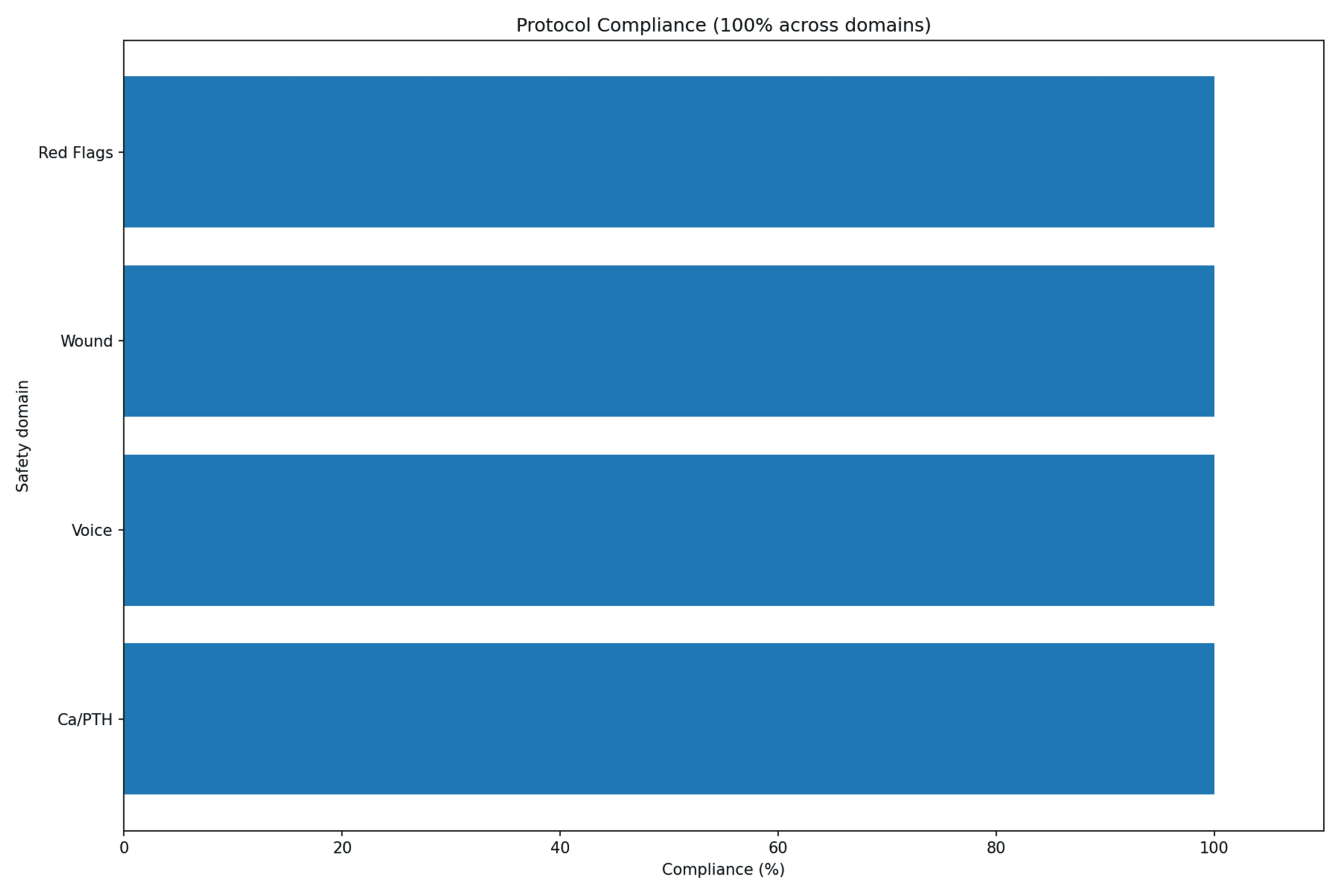
Protocol compliance across safety domains (100%)

Documentation compliance in the nurse-led cohort was 100% for red flag screening, wound assessment, voice documentation, and biochemical review. Compliance across safety domains is illustrated in Figure 2.

Patient satisfaction was uniformly positive with no complaints.

The nurse-led clinic provides 6-7 appointment slots weekly. Following implementation, a visible reduction in pressure on consultant-led head and neck cancer clinics was observed following redistribution of low-risk follow-up.

## Discussion

This retrospective service evaluation indicates that, within a defined low-risk DTC population and a governance-supported pathway, nurse-led follow-up can be delivered safely with a low escalation proportion and high protocol compliance. The approach aligns with principles of dynamic risk stratification [1-3] and with guideline-based emphasis on structured post-treatment monitoring [4].

From a service redesign standpoint, the rationale for stratified follow-up approaches is supported by national cancer follow-up policy advocating reallocation of outpatient capacity and more tailored models of follow-up after cancer treatment [5]. Within this evaluation, the nurse-led clinic was implemented with standardised documentation and explicit escalation routes, consistent with governance safeguards recommended for safe delegation in oncology follow-up models [6,7].

In addition to service efficiency, nurse-led follow-up models may enhance the patient experience through improved continuity of care and increased accessibility to specialist advice [6,7]. Clinical nurse specialists often develop longitudinal relationships with patients undergoing long-term surveillance, which can provide reassurance and improve communication during survivorship care. Within this service evaluation, routine patient feedback during follow-up encounters was uniformly positive, supporting the acceptability of this care model. Nurse-led clinics may also support professional development for specialist nurses by expanding clinical responsibilities, strengthening therapeutic relationships with patients, and promoting greater professional autonomy within clearly defined governance frameworks.

The observed 3.3% escalation proportion (Figure 1) should be interpreted in the context of the small nurse-led cohort (n=30) and the resulting imprecision reflected in the confidence interval. The use of the Wilson score interval is appropriate here because it performs better than simple Wald intervals when event counts are small, providing more reliable interval coverage for single-proportion estimates [9].

Although reported according to principles consistent with quality and service evaluation reporting standards such as SQUIRE 2.0 [10], this study remains a retrospective service evaluation rather than a formal quality improvement study.

This study has several limitations. First, it represents a single-centre retrospective service evaluation, which may limit generalisability to other healthcare settings. Second, the sample size was modest and selected pragmatically for service governance appraisal rather than hypothesis testing, resulting in wide confidence intervals around the escalation estimate. Third, follow-up duration within the evaluation window may not capture very late recurrences typical of differentiated thyroid cancer. Finally, as a service evaluation rather than a controlled comparative study, direct equivalence between nurse-led and consultant-led follow-up cannot be inferred.

## Conclusions

Within a single-centre UK service, a risk-stratified nurse-led thyroid cancer follow-up pathway for carefully selected low-risk DTC patients demonstrated high protocol compliance, low escalation, and no identified short-term safety incidents in this retrospective service evaluation. Standardised documentation, explicit escalation criteria, and local governance oversight remain essential to safe delegation in oncological follow-up care.

## Appendices

Table 1 presents the follow-up proforma used in the present study.

**Table 1:** Nurse-led thyroid cancer follow-up proforma variables

Variable	Description	Data type
Patient ID	Unique anonymised identifier	Numeric
Follow-up date	Date of clinic review	Date
Primary treatment	Thyroidectomy +/- RAI	Categorical
Dynamic risk category	Excellent/indeterminate	Categorical
Thyroglobulin level	Serum Tg value	Numeric
Anti-Tg antibodies	Present/absent	Categorical
TSH level	Serum TSH value	Numeric
Neck ultrasound result	Normal/suspicious	Categorical
Escalation required	Yes/no	Binary
Reason for escalation	Rising Tg/suspicious US/clinical concern/none	Categorical
30-day re-attendance	Yes/no	Binary
